# Circulation autoantibody against Lamin A/C in patients with Sjögren's syndrome

**DOI:** 10.18632/oncotarget.13256

**Published:** 2016-11-09

**Authors:** Wen Zhang, Chunyan Zhang, Peng Chen, Chunhe Yang, Xianfeng Gan, Muhammad Hussain, Yiping Xun, Yaping Tian, Hongwu Du

**Affiliations:** ^1^ 112 Lab, School of Chemistry and Biological Engineering, University of Science and Technology Beijing, Beijing, China; ^2^ Department of Clinical Biochemistry, Chinese PLA General Hospital, Beijing, China

**Keywords:** autoimmune diseases, Sjögren's syndrome, Lamin A/C, autoantigen, similar antigen epitopes, Pathology Section

## Abstract

Lamin A/C proteins are major components of nuclear laminae and were encoded by the LMNA gene. Recent studies have found that in addition to provides nuclear-membrane strength; it also regulates the gene expression. Lamin A/C has been confirmed as an autoantigen in RA, SLE and vasculitis. Anti-Lamin A/C antibodies also have been found by indirect immunofluorescence method. In this study, we used various research methods to confirm Lamin A/C is an autoantigen in Han Chinese patients with confirmed Sjögren's syndrome (SS). To further investigate the relationship between the autoimmune disease antigens, we compared the amino acid sequence of Lamin A/C epitope and several common antigens' antigenic determinant. As a result, we found that Lamin A/C has similar epitopes with U1RNP. It means that the potential relationship exist between Lamin A/C and U1RNP. Clinical data we collected also showed that anti-Lamin A/C and anti-U1RNP antibodies always appear in same serum sample. Therefore, we speculated that cross-reaction may take place between antigen and potential antigen, which have similar epitope. Then, by epitope spreading, the potential antigen can be a new autoantigen. Our study provided a new thinking for further research about the relationship between autoantigens and their development mechanism in autoimmune diseases.

## INTRODUCTION

Sjögren's syndrome (SS) is a systemic autoimmune disease (AID) [1]. Dryness of the eyes and mouth are the main symptoms of SS [2]. Although a lot of important work had been reported in this field, currently the exact mechanism of SS is still unclear [3]. According to AECG [4] and ACR [5] classification criteria, the detection of autoantibodies is an important indicator for SS clinical classification. Anti-Lamin A/C, as a common autoantibody, associated with a variety of systemic symptoms and considered to play a pathological role in some AID pathogenesis like RA [6], systemic lupus erythematosus (SLE) [7] and vasculitis [8].

It is known that Lamin A/C provides nuclear-membrane strength as one of the main proteins of the nuclear envelope. In addition, regulation of gene expression has become its more important function. Lamin A/C interacts with histones and DNA in transcription and replication [9]. Also, it is the cause of laminopathies when mutations in LMNA (gene encodes Lamin A or C) take place [10]. Lots of reports hold the same viewpoint that Lamin A/C mutations could alter the differentiation potential of mesenchymal stem cells (MSCs) [11, 12]. MSCs were found from stromal cells of bone marrow by Friedenstein and colleagues for the first time in 1987 [13]. MSCs differentiate into bone, fat and cartilage. MSCs also contribute to maintain the homeostasis of immunological memory by controlling the memory of T cells and plasma cells in a non-proliferative state [14–16]. Clinical trials are in progress, which are expected to use MSCs treating autoimmunity. In a recent report, in 2015, Lamin A/C acts as an essential factor in MSCs differentiation through the regulation of the dynamics of the wnt/β-catenin pathway and it had been demonstrated [17].

So, it is necessary to find out the significant relationship between Lamin A/C and autoimmune diseases. Since Lamin A/C has been confirmed as an autoantigen in RA, SLE and vasculitis, anti-Lamin A/C antibodies also have been found by indirect immunofluorescence in serum of SS in other studies. The important role of Lamin A/C in SS hadn't been discussed yet [18, 19].

In this study, we examined anti-Lamin A/C antibodies in serum of SS, and discussed the possible relationship between Lamin A/C and other autoantigen of SS. Because, anti-Lamin A/C antibodies have been found in previous research work and the sensitivities of the detection was 50 % [20]. As to better illustrate our results we set serum samples of RA patients as positive control and verified our findings by Immunological methods including Western blotting, immunoprecipitation and ELISA, to prove that Lamin A/C is an autoantigen of SS in Chinese Han population.

## RESULTS

### Lamin A/C protein is an autoantigen of SS

**Figure 1:**
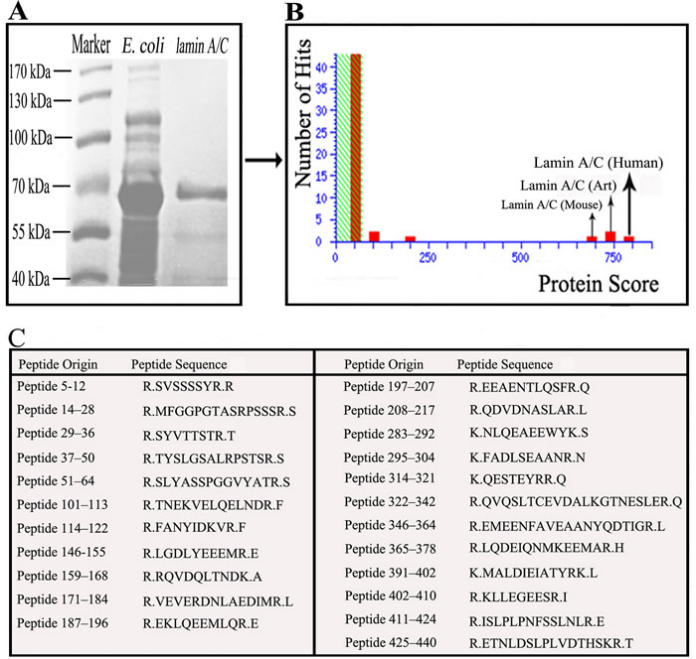
Demonstrate that the 70-kDa protein was pure Lamin A/C **A.** Electrophoresed the bacteria and purify the Lamin A/C, revealed a protein band with about 70-kDa. **B.** Mass spectrum identification revealed that the 70-kDa protein was pure Lamin A/C. **C.** 23 Peptides matched Lamin A/C was identified using mass spectrometry.

**Figure 2:**
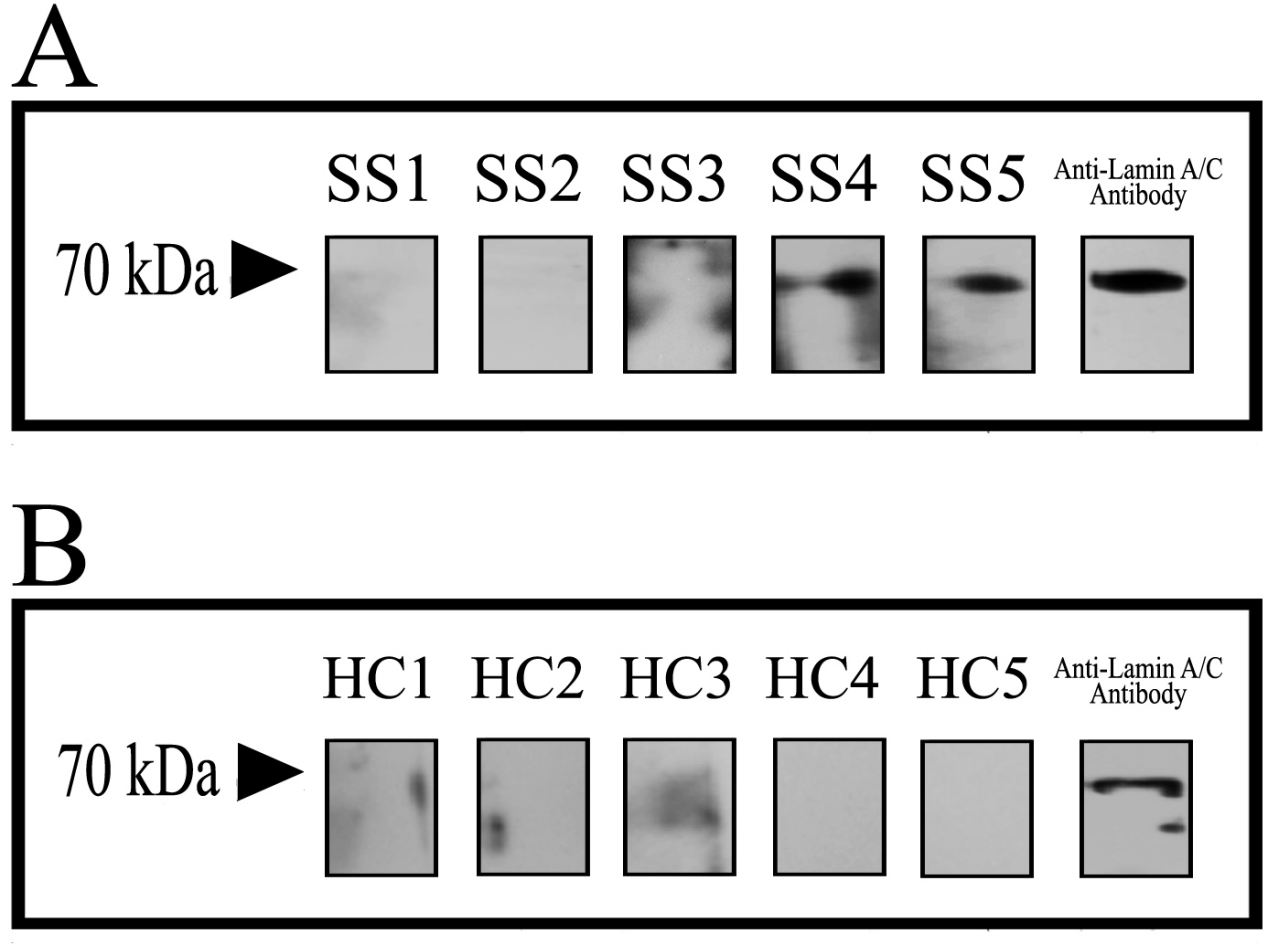
Discover that Lamin A/C is a potential autoantigen of SS 5 serum samples from SS patients **A.** and 5 serum samples from healthy controls **B.** were performed with Western blotting and anti-Lamin A/C antibody in contrast. In SS serum samples 2 of 5 protein bands were detected, but the protein bands were absent in healthy controls.

**Figure 3:**
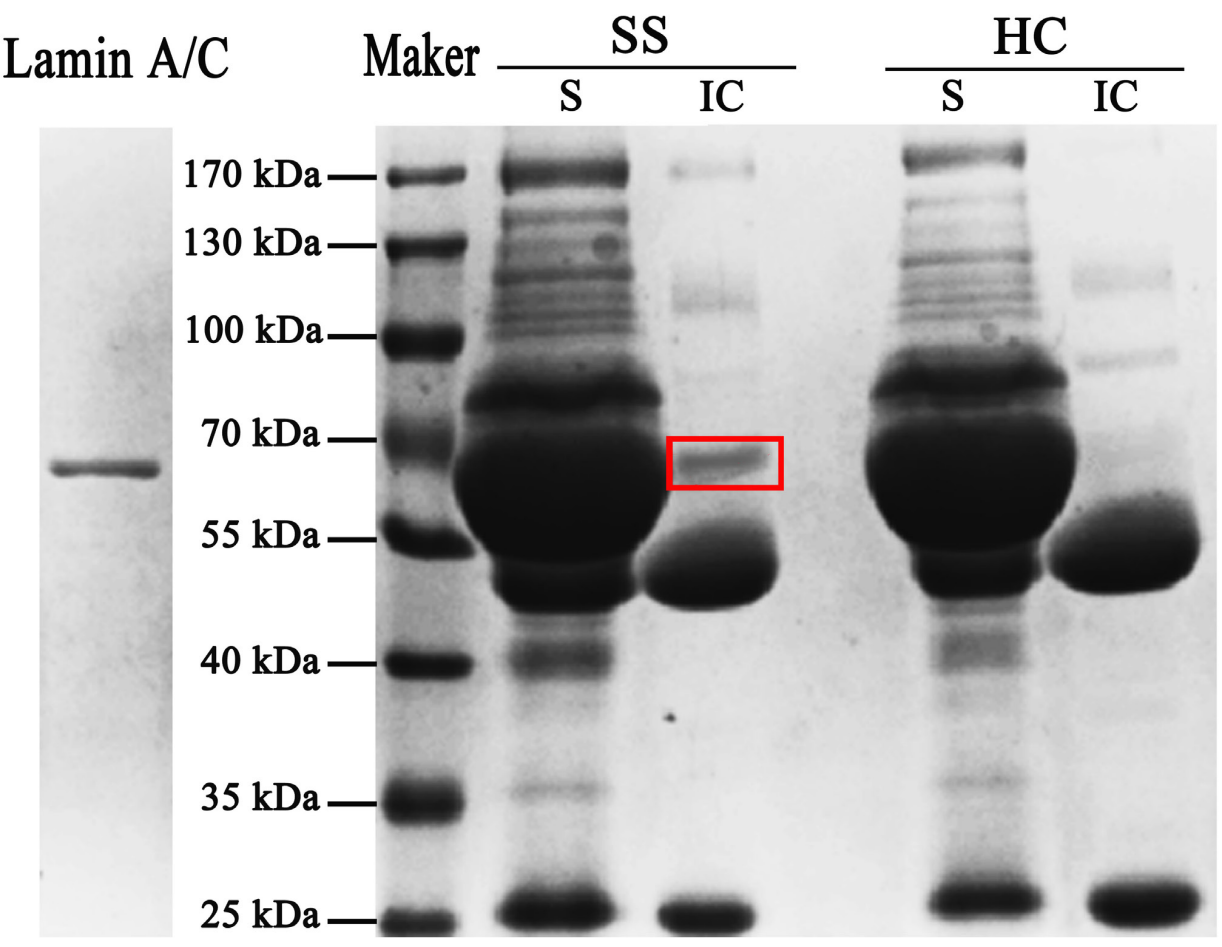
Further prove that Lamin A/C is a potential autoantigen of SS After incubation with the SS and HC sera, the antigen-antibody complexes reacted with protein-A sepharose beads and then electrophoresis was performed with the supernatant (S) and the immune complexes (IC). A protein band of about 70-kDa was present in immune complexes but absent in supernatant.

*E*. coli BL21 transfected with the cloning vectors of Lamin A/C expressed Lamin A/C, after that the protein was collected and purification. Electrophoresis was performed with *E*. coli, transfected with the lamin gene, liquid and purified protein of Lamin A/C. Blots in the middle lane and right lane are electrophoresis results of *E*. coli and purified Lamin A/C, respectively. Mass spectrum identification showed that the 70-kDa protein was pure Lamin A/C (Figure 1) and it means that all the protein used in this test is pure Lamin A/C. 5 serum samples from SS patients (Figure 2A) and 5 serum samples from healthy controls (Figure 2B) were performed with Western blotting. In SS serum samples, 2 of 5 IgG autoantibodies to the 70-kDa protein bands were detected, which were absent in healthy controls. In order to further prove that Lamin A/C is a potential autoantigen of SS, immunoprecipitation was performed, which showed that a protein band of about 70-kDa could react with SS patient sera (Figure 3). Protein band was excised from the gel, digested with trypsin and identified by using MALDI-TOF-TOF mass spectrometer (Applied Biosystems, Foster City, CA). The data was analyzed with Mascot bioinformatics database search engine (Matrix Sciences, London, UK), proving that the target protein was Lamin A/C.

### Prevalence of anti-Lamin A/C antibodies in SS

**Figure 4:**
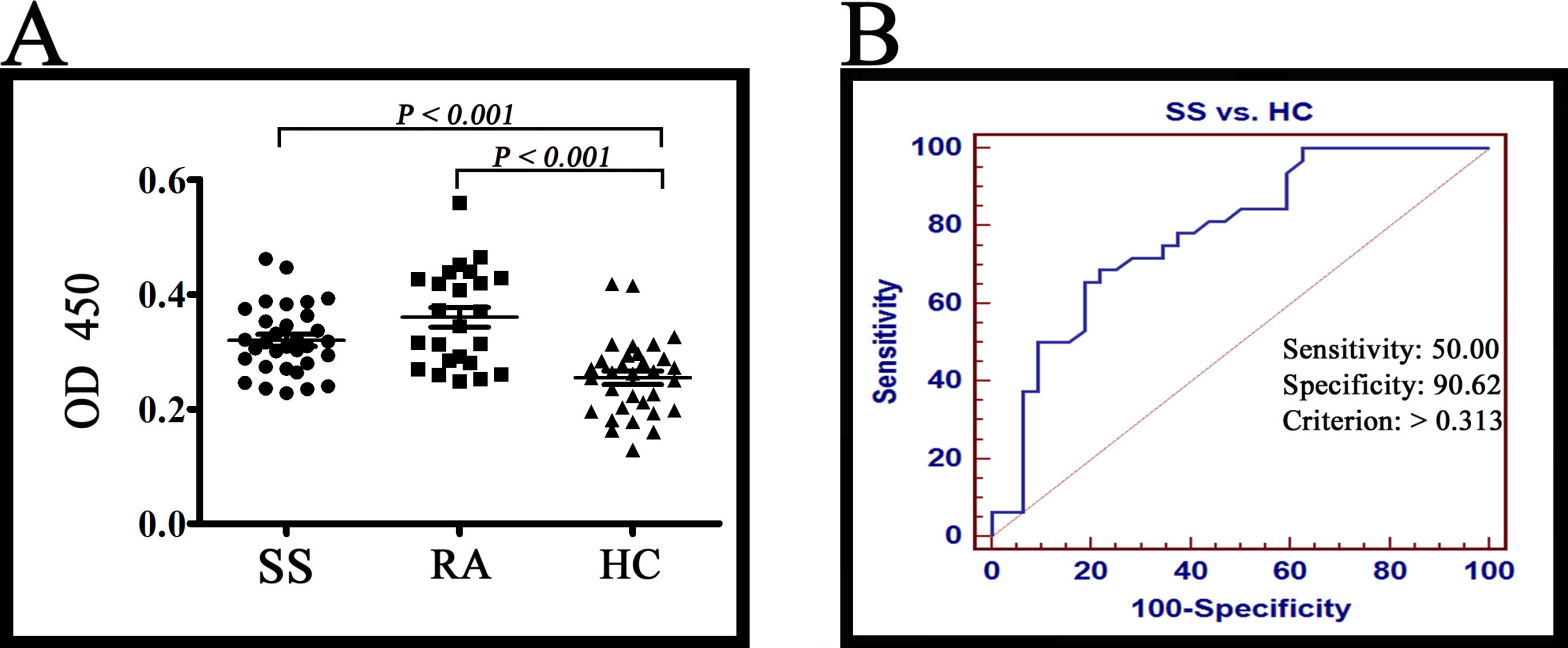
ELISA results **A.** The Lamin A/C was detected in 15 of 24 RA patients (62.5 %), 16 of 32 SS patients (50.0 %) and 3 of 32 healthy controls (9.3 %). The reactivity of SS serum IgG antibodies against Lamin A/C was higher than healthy controls (*P* < 0.001). **B.** ROC analysis was carried out for healthy controls and SS patients with MedCalc (MedCalc Software, Mariakerk, Belgium).

Receiver operating characteristic (ROC) analysis between 32 SS patients and 32 healthy controls was carried out to obtain cut-off positivity, whose results indicate that, at value of 0.313, sensitivity and specificity were 50.00 and 90.62 respectively. Therefore, we set 0.313 as the cut-off value [21]. In the test of ELISA, Lamin A/C was detected in 15 of 24 RA patients (62.5 %), 16 of 32 SS patients (50.0 %) and 3 of 32 healthy controls (9.3 %) (Figure 4). Positive rate of SS was higher than healthy controls (*P* < 0.001). In view of the previous study, Lamin A/C is a confirmed autoantigen in RA [22, 23], so RA was chosen as the positive control. According to results of all tests mentioned above, Lamin A/C is an autoantigen of SS.

### Amino acid sequence similarities between Lamin A/C and other autoantigens

**Figure 5:**
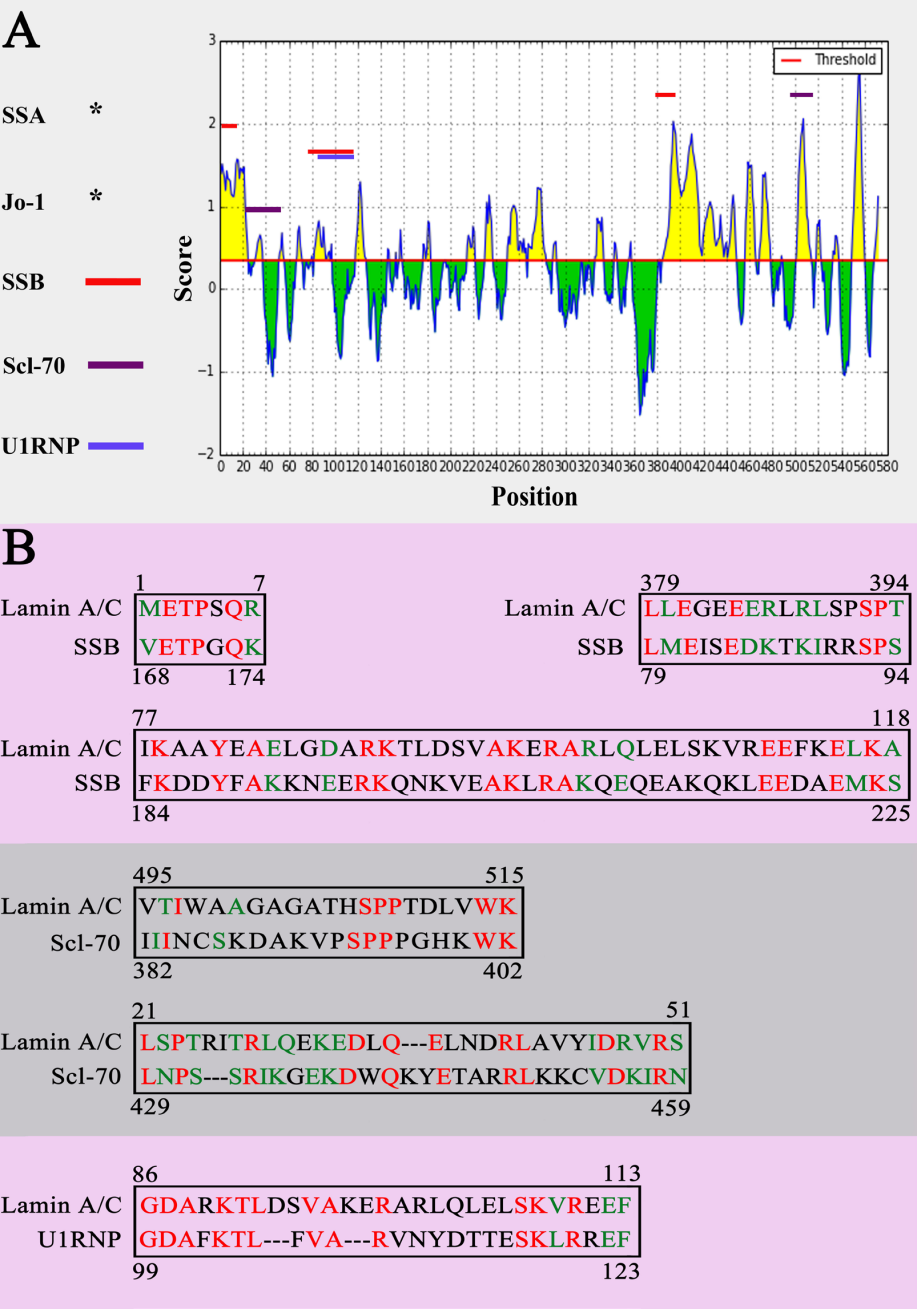
Sequence alignment **A.** The sequence alignment results of Lamin A/C with SSB, Scl-70 and U1RNP. **B.** The same epitopes between potential epitopes of Lamin A/C and antigenic determinants of SSB, Scl-70 and U1RNP. Same amino acid was marked in red. Amino acid with similar properties was marked in green.

According to epitope prediction analysis, eight peptides of Lamin A/C were predicted as potential epitopes, including 1 to 7 (-METPSQR-), 7 to 21 (-RRATRSGAQASSTPL-), 32 to 37 (-KEDLQE-), 80 to 97 (-AYEAELGDARKTLDSVAK-), 250 to 261 (-AQHEDQVEQYKK-), 382 to 394 (-GEEERLRLSPSPT-), 419 to 437 (-RKLESTESRSSFSQHARTS-), 501 to 513 (-GAGATHSPPTDLV-). The results of sequence alignment showed that among eight predicted epitopes some are similar to the confirmed epitopes of SSB, Scl-70 and U1RNP, respectively (Figure 5). SSA/Ro and Jo-1 also have similar sequence with Lamin A/C, but the confirmed epitope of SSA/Ro and Jo-1 doesn't include in the same sequence.

### The situation of autoimmune disease associated antibody presented in patients in ELSA test

**Figure 6:**
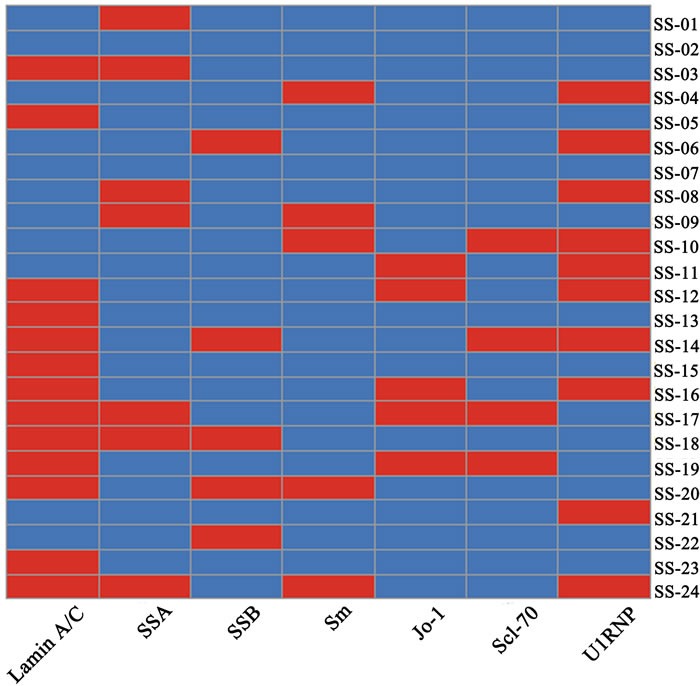
Results of ELISA and clinical test analysis Five antibody clinical test results of SS patients and ELISA result were analyzed. Every rectangle represents one patient and the red denotes positive but the blue negative.

The heatmap of ELISA and clinical tests showed that the disease results between ELISA, SSA/Ro, SSB/La, Sm, Jo-1 and Scl-70 are discrepant but not significant between ELISA and U1RNP (p>0.6). Meanwhile the positive rates of the ELISA result (13/24, 54 %) and U1RNP (10/24, 42 %) for SS both are apparently higher than other groups (Figure 6). These results demonstrated that anti-Lamin A/C antibody and anti-U1RNP antibody were always present in same serum sample at the same time.

## DISCUSSION

In this study, we have provided further evidences and confirmed that Lamin A/C is an autoantigen of SS in Han Chinese patients. Meanwhile some similar antigen epitopes between Lamin A/C and other antigen associated with autoimmune disease were obtained by sequence alignment. Clinical information analysis showed that the presence of anti-Lamin A/C and anti-U1RNP antibody has potential relevancy. This phenomenon may relate the existence of similar antigen epitope between those two proteins.

According to the results of sequence alignment we found that Lamin A/C has similar antigen epitope with SSB/La, Scl-70 and U1RNP. Those similar antigen epitopes may play a very important role in autoimmune response. In 2000, Miura and colleagues [24] found two similar epitopes, ^444^TFYLK^445^, ^458^LCENIAGHLK^467^ and ^445^TFYTK^449^, ^459^LCENIANHLK^468^ in bovine and rat catalase, respectively. It also been confirmed that those two similar epitopes are responsible for cross-reaction. That is to say, cross-reaction occur two proteins with similar epitopes. Thus the autoantibody of SSB/La, Scl-70 or U1RNP may form immune complex with Lamin A/C protein through recognizing similar antigen epitope.

The theory of epitope spreading holds the view that once the immune tolerance of one protein was abrogated, the autoantibody response could diversify via recognition of new epitopes on this protein [25]. Therefore, when antibodies recognizing similar antigen epitope formed immune complex with Lamin A/C protein, epitope spreading could exposed more epitopes, than Lamin A/C became a new target in immune response.

In this study, we also found that the positive rates of ELISA (54 %) and U1RNP (42 %) with 24 patients in clinical information analysis are similar and anti-Lamin A/C antibody can be detected in most of the anti-U1RNP antibody positive serum. The similar phenomenon had been observed about half a century ago. Northway [26] and Mattioli [27] found that autoantibodies of RNP and Sm often were found in the same patient sera. It had been proved that both Sm and RNP were located on the U1 snRNP particle [28] and anti-Sm and anti-RNP antibodies were linked [29]. They are always present in the same individual.

Based on the above analysis of the experimental results and clinical information, we inferred that antibodies of a certain antigen could recognize similar epitope on another potential antigen by cross-reaction. Then epitope spreading let more epitope of potential antigen exposed. Finally, this potential antigen becomes a new autoantigen. Of course, the correctness of this assumption needs further researches to confirm. But keeping in view the above facts and findings, this study provides a new thinking of relationship between autoantigens and their development mechanism in autoimmune diseases.

## MATERIALS AND METHODS

### Serum samples

Two categories of serum samples were collected for this study. In a first category 10 serum samples were collected, five from patients of SS with an average age of 70 years (range 59-77) and 5 serum samples from healthy donors with an average age of 27 years (range 24-30). Samples were analyzed by the Western blotting method (Western blotting group). In a second category (validation group) 32 serum samples from patients with SS with an average age of 55 years (range 18-77), 24 serum samples from patients with RA with an average age of 50 years (range 22-77) and 32 serum samples from healthy people with an average age of 26 years (range 21-32) were taken for ELISA test. All the serum samples were collected from the Chinese PLA General Hospital for present study. The ethical committee of Chinese PLA General Hospital has approved this study and consent was taken from each patient. Serum samples were stored at −80°C for further research.

### Expression and purification of Lamin A/C

The protein expression and purification was performed as described [30].The cloning vector carrying the gene of Lamin A/C was developed as described below.

5′-CAAGAATTCGGTAGCTCCACTCCGCTGT-3′ (Upstream primer)

5;-CCGCCGA GTTATTAATCCTCGTCGTCCTCAAC-3′ (Downstream primer)

The cloning vectors were transferred into *E*.coli BL21 and then the protein of Lamin A/C was expressed efficiently after 6 h of induction with IPTG. Finally the protein was collected in the insoluble fractions of the cell lysates. The soluble supernatant was passed through Ni-NTA resin (Qiagen, Hilden, Germany) and BCA assay kit (Biosynthesis Biotechnology, Beijing, China) and was used to determine the concentration of the protein.

### Mass spectrum identification

Mass spectrum was performed as per reference [31]. In this method the band of pure protein was excised from gel followed by destaining with a mixture of 25 mM NH4HCO3 and 50 % acetonitrile. After drying by vacuum centrifugation, the gel pieces were reduced for 2 h in 10 mM dithiothreitol. As the temperature cool down, 25 mM NH4HCO3 containing 55 mM iodoacetamide, volume was mixed in the same ratio with the dithiothreitol solution and then incubated for 45 min at room temperature in the dark. After covering with 20 μL of the 0.05 M NH4HCO3 buffer with trypsin (Sigma, MO), digestion was performed with the gel pieces overnight at 37 °C. The target proteins were identified using MALDI-TOF-TOF mass spectrometer (Applied Biosystems, Foster City, CA), and the data was analyzed with Mascot bioinformatics database search engine (Matrix Sciences, London, UK).

### Western blotting

The purified Lamin A/C protein was electrophoresed and then transferred to polyvinylidene fluoride membrane (Merck Millipore, MA) according to standard protocols in our lab [32]. After that the membrane was blocked with 5 % nonfat milk in PBS at 37 °C for 1 h. The primary antibody (serum samples from 5 SS patients) and 5 healthy controls were diluted with 1 % nonfat milk at a dilution of 1:1000, at 4 °C for overnight. The membrane was extensively washed 3 times with 5 % PBST buffer before reaction with secondary antibody. Anti-human IgG antibody (ImmunoHunt, Beijing, China) as a secondary antibody was diluted with 1 % nonfat milk in a dilution of 1:10000 at 37 °C for 1 h, followed by washing with 5 % PBST buffer for 3 times again. The Western blotting results were confirmed with enhanced chemiluminescence kit (Applygen, China). The results initially visualized using Gel Doc EZ Imager (Bio-Rad), and then processed using Adobe Photoshop CS3.

### Immunoprecipitation

Immunoprecipitation was performed as per reference [31]. The Lamin A/C (4 mg) was incubated with SS and HC (Healthy Control) sera (equal volumes from 1 SS patients with positive 70-kDa band) at 4 °C for overnight. After that 20 μL protein-A sepharose beads (Sigma, MO) were added at 37 °C. In order to obtain the immune complexes, the solution was centrifuged for 5 min at 5000 rpm. The supernatant was collected from step first and added with 20 μL sample loading buffer, then 30 μL PBS was added and centrifuged for 5 min at 5000 rpm. Finally, 35 μL sample loading buffer was added to the immune complexes. Electrophoresis was performed both, with the supernatant and the immune complexes.

### ELISA Test

The procedure of this method was based on our former work [30, 33]. Lamin A/C protein was added into a 96-well microtiter plate (Corning, NY) and incubated with goat sera overnight at 4 °C. The plate was washed three times using PBST. After that, serum samples were diluted 1:100 with the sample buffer and added in the plate for 1 h at 37 °C. After washing, 100 μL of anti-human IgG (ImmunoHunt, Beijing, China) was added, and then incubated for 1 h at 37 °C. Later, the plate was washed again using PBST, and added with 100 μL TMB solution. After incubation for 10 min at room temperature, 50 μL of 2 M H_2_SO_4_ was added. Finally, the absorbance was measured with a plate reader at 450 nm (Tecan, Hombrechtikon, Switzerland).

### Epitope prediction analysis and protein database search

Confirmed epitopes of SSA/Ro, SSB/La, Sm, Jo-1, Scl-70 and U1RNP were obtained from Immune Epitope Database (IEDB) [34], and antigenic peptides of Lamin A/C were also predicted using BepiPred linear epitope prediction at the immune epitope database analysis resource platform [35]. Available sequence alignment tool blast program at NCBI was used to evaluate their homologous sequences. Then these sequences were checked whether they exist or not in confirmed epitopes of SSA/Ro, SSB/La, Sm, Jo-1, Scl-70 and U1RNP, and predicted antigenic peptides of Lamin A/C.

### Clinical information analysis

The clinical informations of patients in ELISA test were collected from the Chinese PLA General Hospital (Table 1). Among 32 patients in ELISA test, there are 8 patients who haven't been tested for the other autoimmune diseases related antibody. So, there were 24 samples in clinical information analysis. The results of protein concentration test for SSA/Ro, SSB/La, Sm, Jo-1, Scl-70 and U1RNP were confirmed clinically to positive or negative. ELISA test also classify patients into positive or negative. Then the differences of defined results for SS were checked whether the distribution of paired results is significant or not and the heatmap of these results for SSA/Ro, SSB/La, Sm, Jo-1, Scl-70 and U1RNP were obtained in the R statistical programming language.

**Table 1:** The clinical information of Sjögren's syndrome patients (SS) were showed as follow, contained the number of patient whose serum presented anti-SSA, anti-SSB, anti-Sm, anti-Jo-1, anti-Scl-70 and anti-U1RNP antibody confirmed by clinical test

	SS	
**Anti-SSA**	7/32	21.9%
**Anti-SSB**	5/32	15.6%
**Anti-Sm**	5/32	15.6%
**Anti-Jo-1**	5/32	15.6%
**Anti-Sc1-70**	4/32	12.5
**Anti-UIRNP**	10/32	31.2

### Statistical analysis

Fisher's exact test was carried out to evaluate the differences between cohorts with Graph Pad Prism (JMP, Cary, NC). McNemar's test was applied to paired test for consistency among different groups with R statistical programming language. It was considered statistically significant when P values were less than 0.05. ROC curve analysis was performed between SS patients and healthy controls with MedCalc 9.2 (MedCalc Software, Belgium).
